# Health-related quality of life after emergency abdominal surgery

**DOI:** 10.1186/s13017-025-00643-1

**Published:** 2025-09-02

**Authors:** Vladimir Sergeevich Gordeev, Esubalew Assefa, Rupert Pearse, Mark Edwards, Borislava Mihaylova

**Affiliations:** 1 Health Economics and Policy Research Unit, Wolfson Institute of Population Health, Queen Mary University of London, Yvonne Carter Building, 58 Turner Street, London, E1 2AB UK; 2 Faculty of Medicine and Dentistry, William Harvey Research Institute, Queen Mary University of London, London, UK; 3 Anaesthesia and Critical Care Research Unit, University Hospital Southampton NHS Foundation Trust, Southampton, UK; 4 Acute, Critical & Perioperative Care Research Group, NIHR Biomedical Research Centre, University Hospital Southampton NHS Foundation Trust/the University of Southampton, Southampton, UK; 5 Health Economics Research Centre, Nuffield Department of Population Health, University of Oxford, Oxford, UK

**Keywords:** Health-related quality of life, Emergency abdominal surgery, Prediction modelling, Post-surgery outcomes, Surgery

## Abstract

**Background:**

Patients’ survival and quality of life are key factors in assessing value of treatments. However, limited evidence exists about the trajectory and key determinants of patients’ health-related quality of life (QoL) following emergency abdominal surgery.

**Methods:**

Using the Enhanced Peri-Operative Care for High-risk patients study with measured QoL during eight months follow-up using the EQ-5D-3L questionnaire, we summarise the trajectory of patients’ QoL after emergency abdominal surgery and use multivariable regression models to relate patients’ demographic and clinical characteristics, pre-surgery characteristics, and time elapsed since surgery with their QoL. In further analysis we assess the contribution of post-surgery patient characteristics.

**Results:**

Data from 686 patients undergoing emergency abdominal surgery (50.4% female; mean age 66.6 (standard deviation (SD) 12.8) years; 50.1% with intestinal obstruction as indication for surgery), with QoL measurements were analysed. Shortly after surgery (mean days 7.59 (SD 7.48)), the mean EQ-5D-3L QoL utility score was 0.21 (SD 0.46), which improved among survivors to 0.74 (SD 0.31) in the medium- to long-term (i.e., three to eight months) following surgery. Patient’s sex and preoperative risk of mortality were key determinants of QoL shortly after surgery. In addition to time since surgery, patient’s sex, Charlson Comorbidity index, ASA physical status and indication for surgery were key pre-surgery predictors of QoL in the medium- to long-term post-surgery. From post-surgery characteristics, duration of hospital admission for index surgery and further days in hospital within 30 days prior to QoL measurement were key further determinants of QoL in the medium- to long-term.

**Conclusions:**

Individual patient, surgery, and recovery characteristics determine QoL post-emergency abdominal surgery and can help inform clinician-patient discussions and assessments of value of abdominal surgery interventions.

**Supplementary Information:**

The online version contains supplementary material available at 10.1186/s13017-025-00643-1.

## Background

Emergency abdominal surgery is a resource-intensive, time-critical, high-risk surgical operation to treat acute intra-abdominal conditions. It is associated with a high risk of morbidity and mortality, poor postoperative outcomes, complications, and prolonged hospital length of stay [1, 2]. Worldwide, among emergency abdominal surgery patients, the reported 30-day mortality rates are up to 14.3% [3–5] and one year mortality ranged from 15.1 to 47% [6]. In England and Wales, more than 30,000 patients undergo emergency abdominal surgery annually [7]. The National Institute for Health and Care Excellence (NICE) guideline for perioperative care in adults covering elective and emergency surgery stipulates that patients have the right to discuss and make informed decisions about their care and express their personal needs and preferences [8]. Health-related quality of life (QoL) is identified as a core outcome in perioperative care to capture patients’ longer term recovery post-surgery [9] and post-surgery QoL has been shown to be an important outcome patients valued, even above risk of mortality, in their decision to undergo emergency surgery [10]. Hence, in addition to typical immediate clinical outcomes (wound healing, infections, other complications, and survival), clinicians need to communicate to patients the likely trajectory of their QoL after surgery. For example, will the patient be able to perform routine activities as before the surgery, take care of themselves, and remain mobile, or will they experience pain, discomfort, or anxiety? Preparing and empowering patients, and facilitating a meaningful discussion pre- and post-surgery, could help manage patients’ expectations about the recovery process and life after surgery.

Previous studies have shown that the outcomes following emergency abdominal surgery varied by pre- and post-surgery characteristics, such as pathology, indication for surgery, perioperative sepsis, dependency status, comorbidities, age, frailty, American Society of Anaesthesiologists (ASA) physical status grade and hospital admission length of stay (LOS) [11–14]. Diabetes mellitus, smoking, osteoarthritis, and malignancy were also associated with lower QoL following gastrointestinal surgery [15, 16]. Surgical complications [17, 18] were associated with lower QoL post-surgery, and transfer to intensive care unit (ICU) after surgery (compared to regular ward) was associated with increased mortality [4].

The immediate and short-term outcomes post-emergency abdominal surgery (i.e., mortality, adverse events, complications, and length of stay) are well established and recorded routinely. However, the broader health outcomes post-emergency abdominal surgery from a patient’s perspective, including QoL, are largely unknown [19]. A recent systematic review of studies assessing the economic impact of emergency abdominal surgery showed that in-hospital and 30-day mortality, morbidity, hospital LOS or a stay in an intensive care unit (ICU), and associated costs, but not health-related QoL measures were the most frequently used and studied outcome measures [20]. Moreover, emergency abdominal surgery complications can extend beyond the immediate postoperative period [21], confirming a need to examine the longer-term impact of emergency abdominal surgery on patients’ health-related QoL. This study aimed to (1) describe patients’ QoL trajectory post-emergency abdominal surgery, and (2) identify key patient sociodemographic and clinical characteristics determining their QoL post-emergency abdominal surgery.

## Methods

### Study data

We performed secondary retrospective analyses using individual patient data from the Enhanced Peri-Operative Care for High-risk patients (EPOCH) trial [22]. EPOCH was a multicentre, stepped-wedge cluster-randomised study of the effectiveness of a national quality improvement programme to improve survival within 90 days among 40 years and older patients who underwent emergency abdominal surgery in 93 hospitals in the United Kingdom (UK), participating in the National Emergency Laparotomy Audit (NELA) [23]. The EPOCH trial took place in 2014–2016 and was approved by the East Midlands (Nottingham 1) Research Ethics Committee (reference number 13/EM/0415). No survival benefit was observed from the quality improvement programme in the study [22]. Patient characteristics and surgical treatment data were extracted from the NELA database [23], hospital admissions from the Hospital Episodes Statistics (HES) Admitted Patient Care (APC) [24] and mortality data from the Office for National Statistics (ONS) death registry [25] using participants’ unique identifiers.

**Fig. 1:**
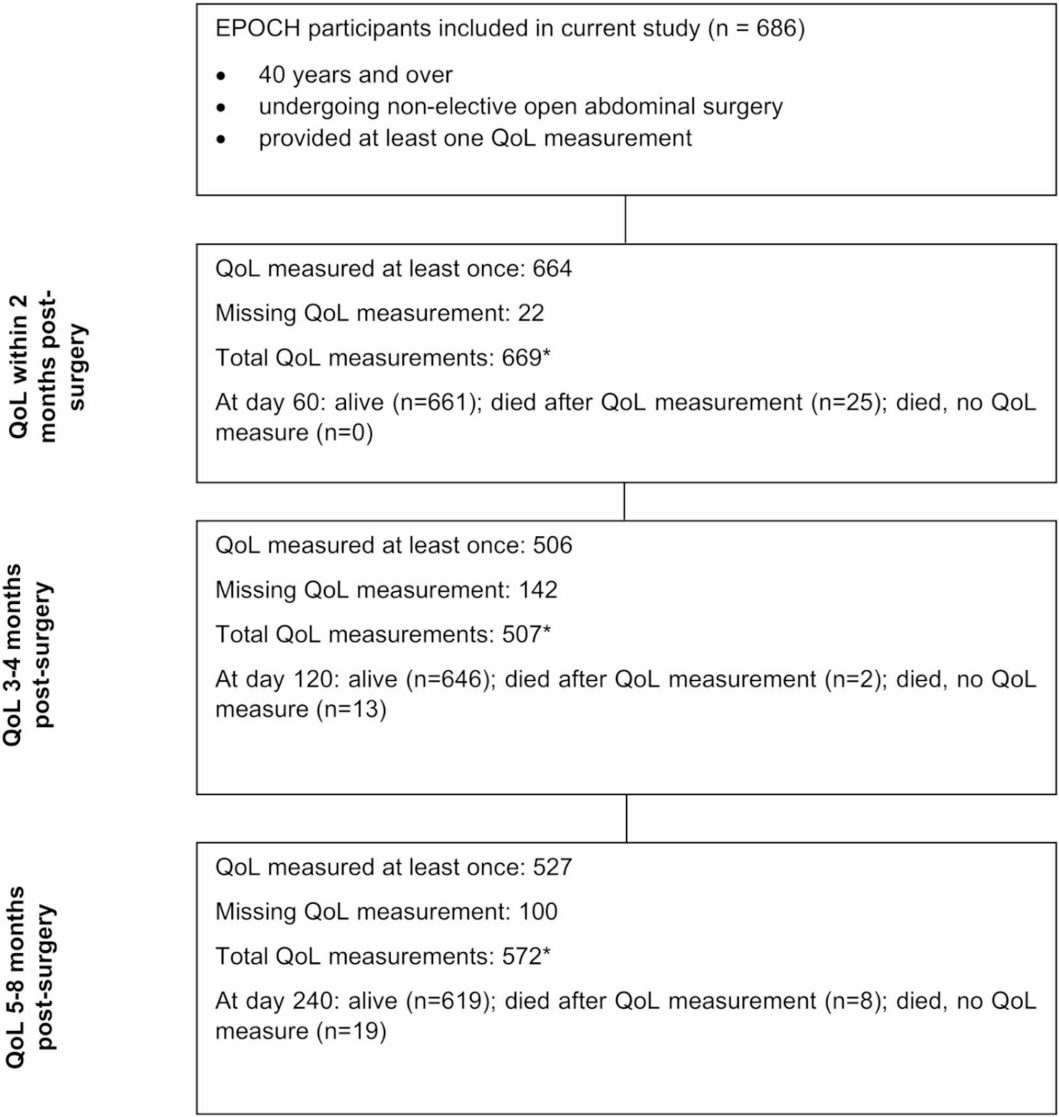
Study population and quality of life measurements. QoL, quality of life; EPOCH, Enhanced Peri-Operative Care for High-risk patients study. *some participants provided more than one EQ-5D-3L measurement during the respective period. Further details presented in Figure S2, Supplementary Material 1

In eight of the 93 hospitals in the EPOCH study, QoL data was collected from eligible patients at three time points: shortly after the index emergency abdominal surgery (short-term, within 1–2 months), and during the first (medium-term, 3–4 months, at about 90 days) and second (long-term, 5–8 months, at about 180 days) post-surgery follow-ups among survivors (Fig. 1; Figures S1-S2; Supplementary Material 1).

### Quality of life measurement

Health-related QoL in EPOCH was measured using the EQ-5D-3L questionnaire [26], which includes questions related to five dimensions: mobility, self-care, usual activities, pain/discomfort, and anxiety/depression; each measured using three levels of responses (no; some; and extreme problems/unable). Patients’ EQ-5D-3L responses were converted into QoL utility scores using the UK tariff [27] on a scale from 1 (full health) to -0.594 (worse than death health state), where 0 is a health state equivalent to death.

### Patient characteristics

We assessed the impact of a set of demographic and clinical pre- and post-surgery characteristics on QoL, informed by the literature and data availability in the EPOCH trial. The following preoperative factors were included: patient’s sex and age at admission, clinical indication for surgery, preoperative ASA physical status grade, comorbidities and preoperative risk of mortality. Age was categorised into < 60 years old, 60 to 69, 70 to 79 and 80 years or over. Indication for surgery was categorised into obstruction, haemorrhage, sepsis, ischemia and other following NELA’s technical guide [28]. Comorbidities were specified using Charlson comorbidity index (CCI) [29, 30] (i.e. none; mild (score 1–2); moderate (score 3–4); and high risk (score ≥ 5)), based on 17 co-morbid conditions (Table S1, Supplementary Material 1). Preoperative risk of mortality had three levels: low (< 5%), high ( > = 5%) and not documented. Additionally, we assessed the role of the following surgery and post-surgery factors: surgery location, length of hospital stay after surgery, initial postoperative care in ICU, duration between index surgery and QoL measurement and days in hospital within 30 days prior to QoL measurement. The surgery location was categorised as small intestine, large intestine, stomach or other. We established the duration between the index surgery and each QoL assessment post-surgery. Using HES data, we established whether participants were readmitted post-index surgery to the hospital in the 30 days before each postoperative QoL measurement and calculated cumulative LOS during these 30 days excluding index surgery.

### Statistical analysis

First, we estimated a multivariable linear regression model (model 1) of patients’ short-term QoL (one to two months) after surgery using patient’s sex, age, comorbidities, ASA, preoperative risk of mortality and indication for surgery. Second, we estimated two models of medium- to long-term QoL (three to eight months after surgery). Initially, we estimated a multivariable linear regression model of medium- to long-term QoL using the same patient characteristics as in model 1 together with the duration between index surgery and patient’s QoL measurement (model 2a). Subsequently, we extended this model by adding the following post-surgery factors: surgery location, postoperative care in ICU, index surgery LOS and days in hospital within 30 days prior to QoL measurement (model 2b). Standard errors were clustered by patient to account for multiple QoL observations per patient. The final models were estimated following multiple imputation of missing values. Multiple imputation by chained equations with the full set of outcomes and predictors informing the QoL models was used to impute missing QoL observations and predictors [31]. Predictive mean matching was used for the EQ-5D-3L utility score, with the number of closest observations set at five. Logistic and multinomial logistic regression methods were used for binary and categorical variables with missing values. Twenty imputed data sets were generated, analysed, and results summarised using Rubin’s rule, implemented using the *‘mi estimate’* command in Stata. In a sensitivity analysis, the models were re-estimated using the complete case data only. All analyses were performed using Stata 18 [32].

### Validation and applications

We applied the results of the short-term and medium- to long-term models to predict QoL of participants. We compared the predicted mean QoL with patient-reported QoL by age, sex, and CCI categories in respective periods. To account for parameter uncertainty in predictions, we sampled 1000 sets of coefficients using the final regression models, assuming a multivariable normal distribution and employing the Cholesky decomposition of the covariance matrix to preserve correlations [33]. We additionally presented patients’ QoL trajectories post-index emergency abdominal surgery in EPOCH alongside the QoL of patients who underwent elective abdominal surgery (using data from the Prevention of Respiratory Insufficiency after Surgical Management (PRISM) trial [34] and alongside the QoL reported by the general UK population of similar age and sex using data from Health Survey for England [35].

## Results

The characteristics of the 686 emergency abdominal surgery patients in the EPOCH study contributing to the present study are summarised in Table 1. A total of 664 (96.8%) patients responded to the QoL questionnaire after index emergency abdominal surgery (mean days from surgery 7.59 (SD 7.48)), 506 (78% from eligible patients) by the end of the first, and 527 (84.1% from eligible patients) by the end of the second follow-up period (Fig. 1). The mean (SD) age of study participants at the time of surgery was 66.6 (12.8) years, ranging from 40 to 92 years, and 346 (50.4%) were female (Table 1). Obstruction 343 (50.1%) was the most frequent indication for index surgery, followed by sepsis 221 (32.3%). The ASA-grade physical status of patients was mainly mild or severe systemic with no life-threatening disease, 234 (34.1%) and 272 (39.6%), respectively. A larger proportion of participants, 504 (73.5%), were admitted to ICU post-surgery compared to those admitted to ward recovery. The mean (SD) length of hospital stay after surgery was 22.1 days (24.3) and the median was 15 days (IQR 10 to 24).

**Table 1:** Characteristics of study participants

	Up to 2 months post-surgery (*n* = 686)			3 to 8 months post-surgery (*n* = 648)	
	N	(%)		N	(%)
**Sex**					
Male	340	(49.6)		322	(49.7)
Female	346	(50.4)		326	(50.3)
**Age**,** Mean (SD)**	66.6	(12.8)		66.1	(12.8)
< 60 years	209	(30.5)		208	(32.1)
60–69 years	166	(24.2)		157	(24.2)
70–79 years	180	(26.2)		166	(25.6)
80 + years	131	(19.1)		117	(18.1)
**Comorbidity**,** Charlson Comorbidity Index**					
No comorbidity	300	(46.4)		284	(46.7)
Mild	256	(39.6)		244	(40.1)
Moderate	66	(10.2)		57	(9.4)
Severe	24	(3.7)		23	(3.8)
**ASA physical status**					
Normal healthy patient/ Mild systemic disease	288	(42.0)		283	(43.7)
Severe systemic disease	272	(39.7)		253	(39.0)
Severe systemic disease, life threatening	116	(16.9)		102	(15.7)
Moribund	10	(1.5)		10	(1.5)
**Preoperative risk of mortality**					
Low (< 5%)	157	(22.9)		152	(23.5)
High ( > = 5%)	271	(39.5)		251	(38.7)
Not documented	258	(37.6)		245	(37.8)
**Indication for surgery**					
Haemorrhage	19	(2.8)		19	(2.9)
Obstruction	343	(50.1)		318	(49.1)
Sepsis	221	(32.3)		216	(33.4)
Ischaemia	69	(10.1)		65	(10.0)
Other	33	(4.8)		29	(4.5)
**Index surgery location**					
Large intestine	242	(35.3)		233	(36.0)
Small intestine	125	(18.2)		113	(17.5)
Small or large intestine	36	(5.3)		32	(4.9)
Stomach	55	(8.0)		52	(8.0)
Other (abdominal cavity/wall)	227	(33.1)		217	(33.5)
**Discharge destination after index surgery**					
Ward	182	(26.5)		172	(26.5)
ICU/HDU	504	(73.5)		476	(73.5)
**Length of hospital stay after surgery**,** Mean (SD)**	22.1	(24.3)		21.8	(24.6)
<= 10 days	188	(27.4)		182	(29.9)
11–14 days	136	(19.8)		89	(14.6)
15–21 days	153	(22.3)		149	(24.5)
21 + days	209	(30.5)		188	(30.9)
**Participants with QoL measurement**	664	(96.8)		506	(78)
**Reported QoL measurements**	669*			1079*	
**QoL Index**,** Mean (SD)**	0.21	(0.46)		0.74	(0.31)
**Length of hospital stay within 30 days before QoL measurement (days)**,** Mean (SD)**				0.66	(2.87)
**Duration between index surgery and QoL measurement (days)**,** Mean (SD)**	7.59	(7.48)			
1–4 days	247	(36.9)			
5–7 days	204	(30.5)			
> 1 week	218	(32.6)			

ASA, American Society of Anesthesiologists; QoL, quality of life

* Some participants provided more than one EQ-5D-3L measurement during the respective period

### Patients’ health-related QoL trajectories post-emergency abdominal surgery

**Fig. 2:**
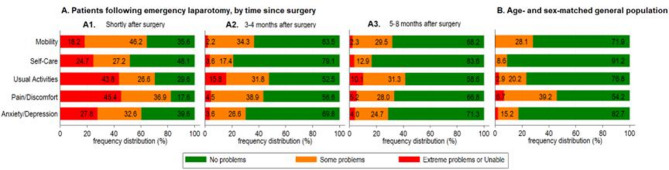
Quality of life after emergency abdominal surgery compared with age- and the sex-matched general population. EQ-5D-3L QoL descriptive system comprises five domains: mobility, self-care, usual activities, pain/discomfort, and anxiety/depression. Each domain has three levels: no, some, and extreme problems. General population: 2-to-1 age- and sex-matched sample drawn from the Health Survey for England participants (2014) [35]

The mean QoL shortly after surgery (up to 2 months) was 0.21 (SD 0.46). 304 (45.4%) and 293 (43.8%) patients reported extreme pain/discomfort and extreme problems with usual activities, respectively (Fig. 2A). At least some problems were reported by 431 (64.4%) patients for mobility, 347 patients (51.9%) for self-care, and 404 (60.4%) for anxiety or depression. QoL improved in the medium- to long-term with mean QoL from 3 to 8 months post-surgery of 0.74 (SD 0.31). As the time duration from index surgery increased, participants reported having fewer problems across all QoL domains with increasing proportion of participants reporting no problems in all five of the EQ-5D domains, coming closer to the average QoL reported by the age- and sex-matched general 2014 UK population (based on Health Survey for England [35], matched 2:1 to EPOCH participants) (Fig. 2B).

### Determinants of health-related QoL post emergency abdominal surgery

The short-term QoL model (Table 2, Model 1) indicated that sex and preoperative risk of mortality were significantly associated with QoL shortly after surgery. Female patients reported 0.118 lower QoL (SE 0.036) than male patients. Similarly, compared to participants assessed to be at low risk of mortality preoperatively, those with high risk and not documented risk reported lower QoL (-0.114 (SE 0.052), and − 0.120 (SE 0.046), respectively).

**Table 2:** Health-related quality of life after emergency abdominal surgery: multivariable linear regression models

	Model 1: Short-term QoL (up to 2 months post-surgery)			Model 2a: Medium- to long-term QoL (3 to 8 months post-surgery) with patient and clinical pre-op characteristics			Model 2b: Medium- to long-term QoL (3 to 8 months post-surgery) including post-op characteristics		
	691 observations from 686 participants			1321 observations from 648 participants			1321 observations from 648 participants		
	Coef.	(SE)	*p*-value	Coef.	(SE)	*p*-value	Coef.	(SE)	*p*-value
**Constant**	0.387	(0.050)^a^	< 0.001	0.791	(0.031)^b^	< 0.001	0.832	(0.036)^c^	< 0.001
**Sex (Reference: male)**									
Female	-0.118	(0.036)	0.001	-0.057	(0.021)	0.008	-0.047	(0.021)	0.02
**Age (Reference: <60 years)**			0.06^d^			0.27^d^			0.26^d^
60–69 years	0.014	(0.050)	0.78	0.023	(0.028)	0.42	0.030	(0.027)	0.26
70–79 years	0.078	(0.051)	0.13	0.012	(0.031)	0.70	0.024	(0.030)	0.42
80 + years	0.087	(0.056)	0.12	0.047	(0.036)	0.19	0.041	(0.035)	0.23
**Comorbidity**,** Charlson Comorbidity Index (Reference: No comorbidity)**			0.03^d^			0.03^d^			0.05^d^
Mild	-0.066	(0.041)	0.11	-0.033	(0.024)	0.17	-0.033	(0.024)	0.18
Moderate	-0.110	(0.065)	0.09	-0.098	(0.049)	0.05	-0.111	(0.048)	0.02
Severe	-0.131	(0.098)	0.19	-0.072	(0.074)	0.33	-0.041	(0.071)	0.57
**ASA physical status (Reference: Normal healthy patient/ Mild systemic disease)**			0.99^d^			0.005^d^			0.03^d^
Severe systemic disease	0.023	(0.045)	0.61	-0.071	(0.026)	0.007	-0.057	(0.026)	0.03
Severe systemic disease, life threatening	-0.014	(0.062)	0.82	-0.121	(0.040)	0.003	-0.094	(0.040)	0.02
Moribund	0.040	(0.133)	0.76	-0.056	(0.102)	0.59	-0.038	(0.095)	0.69
**Preoperative risk of mortality (Reference: Low (< 5%))**			0.03^d^			0.12^d^			0.26^d^
High ( > = 5%)	-0.114	(0.052)	0.03	-0.041	(0.031)	0.19	-0.017	(0.030)	0.56
Not documented	-0.120	(0.046)	0.01	0.016	(0.026)	0.54	0.025	(0.026)	0.33
**Indication for surgery (Reference: Obstruction)**			0.42 ^d^			0.05 ^d^			0.36 ^d^
Haemorrhage	0.062	(0.115)	0.59	0.001	(0.060)	0.99	-0.031	(0.059)	0.61
Sepsis	-0.069	(0.041)	0.09	0.052	(0.023)	0.02	0.031	(0.025)	0.22
Ischaemia	-0.065	(0.063)	0.31	-0.028	(0.043)	0.51	-0.018	(0.041)	0.66
Other	-0.018	(0.077)	0.82	-0.065	(0.056)	0.25	-0.046	(0.053)	0.38
**Duration between index surgery and QoL measurement (Reference: 3–4 months)**						< 0.001^d^			0.007^d^
5–6 months				0.004	(0.034)	0.91	0.016	(0.031)	0.60
7–8 months				0.059	(0.016)	< 0.001	0.045	(0.016)	0.006
**Index surgery location (Reference: Large intestine)**									0.04 ^d^
Small intestine							0.009	(0.030)	0.76
Small or large intestine							-0.080	(0.055)	0.15
Stomach							0.073	(0.035)	0.04
Other (abdominal cavity/wall)							-0.037	(0.026)	0.16
**Discharge destination after index surgery (Reference = Ward)**									
ICU/HDU							-0.009	(0.024)	0.71
**Length of hospital stay after surgery (Reference: <=10 days)**									0.002^d^
11–14 days							-0.002	(0.029)	0.96
15–21 days							-0.030	(0.027)	0.26
21 + days							-0.090	(0.030)	0.003
**Length of hospital stay within 30 days before QoL measurement**							-0.024	(0.005)	< 0.001
R-squared	0.047			0.077			0.143		
Prob > F	0.01			< 0.001			< 0.001		

^a^ Short-term QoL for a reference patient: a male patient of age 40–59 years, assessed to be ASA class I or II (normal healthy or mild systemic disease) with low (< 5%) preoperative risk of mortality and obstruction as indication for surgery

^b^ Medium- to long-term QoL for a reference patient: a male patient of age 40–59 years, assessed to be ASA class I or II (normal healthy or mild systemic disease) with low (< 5%) preoperative risk of mortality, obstruction as indication for surgery and 3–4 months post-surgery

^c^ Medium- to long-term QoL for a reference patient: a male patient of age 40–59 years, assessed to be of ASA class I or II (normal healthy or mild systemic disease), with low (< 5%) preoperative risk of mortality, obstruction as indication for surgery, main procedure performed on large intestine, discharged to ward post-surgery, stayed 10 days or less in hospital during index surgery, no hospitalisation within 30 days before QoL measurement and 3–4 months post-surgery

^d^*p*-value for linear trend test if ordinal or heterogeneity test if nominal variables

R-squared based on Fisher’s z transformation (using ‘mibeta’ Stata command)

QoL, quality of life; Prob, probability; F, F-value

The medium- to long-term QoL model using pre-surgery characteristics only also indicated that female sex was associated with lower QoL (-0.056; SE 0.021) (Table 2, Model 2a). Worse physiological status, as assessed by ASA physical status classification, was another factor associated with lower QoL in the medium/longer term (*trend p* = 0.0045). Increased duration since surgery was also associated with improved QoL (*trend p* = 0.0003). Compared to 3–4 months from surgery, QoL from 6 months onwards was improved (0.059 (SE 0.016)).

Following the inclusion of post-surgery factors into the medium- to long-term QoL model, female sex (compared to reference group male) (-0.047 (SE 0.021)), worse ASA-assessed physiological status (*trend p* = 0.0281) remained associated with lower QoL. In addition, longer LOS after index surgery (over 21 days compared to LOS ≤ 10 days: -0.090 (SE 0.030); *trend p* = 0.0023) and length of stay in hospital within 30 days before QoL measurement (-0.024 (SE 0.005) per day) were also associated with lower QoL (Table 2, Model 2b). There was improvement in QoL as time passes since surgery (*trend p* = 0.0065) where QoL at 7–8 months improved by 0.045 (SE 0.016) compared to 3–4 months.

There were no differences in baseline characteristics between patients who completed QoL assessments and those who did not at short-term and medium- to long-term follow-up assessments, respectively (Table S2, Supplementary Material 1). In sensitivity analyses, the estimates derived using complete cases only were consistent with models estimated using multiply imputed data (Table S3, Supplementary Material 1). In internal QoL models’ validation, model-predicted QoL corresponded well to reported QoL in short-term and medium- to long-term after surgery in categories of patients by sex, age, and comorbidities (Table S4, Supplementary Material 1). QoL improved substantially from short-term to medium- to long-term in all these categories of patients, although QoL was lower at medium- to long-term in women, people 70 years or older, and those with moderate or severe comorbidities before surgery.

An Excel calculator for the implementation of the health-related QoL models accompanies the manuscript (Supplementary Material 2).

## Discussion

This assessment of patients’ health-related QoL after emergency abdominal surgery indicated improving QoL over time with patients reporting fewer problems across all QoL domains and their QoL approaching the average age- and-sex specific QoL reported in the general population. We report that female sex and high preoperative mortality risk are associated with lower QoL immediately after surgery. In the medium- to long-term, indication for surgery, comorbidities, preoperative physical status grade, days in hospital for index surgery and further days in hospital importantly determine patients’ QoL. We proposed QoL models to predict patients’ QoL trajectories using their demographic and clinical characteristics that can aid doctor-patient discussions of expected recovery process and life after surgery.

As the time elapsed since surgery increases, surviving patients report fewer health problems and better QoL irrespective of their sex, age or comorbidities. This is the case for both emergency abdominal surgery patients in EPOCH and patients who underwent elective abdominal surgery in PRISM. Both emergency and elective abdominal surgery patients reported health issues, predominantly related to pain/discomfort and problems with usual activities, followed by problems with mobility and mental well-being (Figure S3, panel B, Supplementary Material 1). However, a greater magnitude of health problems was reported by emergency abdominal surgery patients throughout their recovery post-surgery, possibly reflecting the higher severity of emergency abdominal surgery and related complications. Nevertheless, in both populations, QoL improved over time and approached the average QoL reported by the general UK population of similar age and sex (Fig. 2).

Quality of life was amongst the most important outcomes professionals involved in the care of emergency abdominal surgery patients wished to predict [36]. However, it is not measured routinely and studies assessing QoL of patients post emergency abdominal surgery are scarce [19]. This study provides evidence on the trajectory of quality of life after emergency surgery using large individual participant data.

Compared to elective surgery, there are challenges to patient and family education and shared decision-making in emergency settings [37, 38]. While acknowledging the challenges, Enhanced Recovery After Surgery (ERAS) Society Guidelines for Perioperative Care for Emergency Laparotomy recommends discussion among patients and families with a senior physician prior to surgery [37]. The guidelines suggest the use of objective mortality scores complemented with other assessments such as frailty scores to support the discussion and encourages the conversations to include other factors such as quality of life. The World Society of Emergency Surgery (WSES) position paper similarly outlined the importance of patient education and counselling and encouraged its implementation to explain risks preoperatively [38]. The models developed in our study allow estimating patents’ QoL shortly after (up to two months) and in a medium- to long-term (3 to 8 months) after surgery providing a systematic approach to predict QoL using preoperative demographic and clinical patient characteristics as well as operative and postoperative variables. These predictions could provide a useful tool to characterise patents’ expected QoL informed by their characteristics and planned operative aspects, which, in turn, can generate an essential input to facilitate discussions and plan care for patients undergoing emergency surgery. To assist such efforts, an Excel program for the implementation of the health-related QoL models is available with the manuscript (Supplementary Material 2).

In this study, QoL was assessed with EQ-5D, a brief generic preference-based QoL measure widely used across different health conditions and interventions, including in evaluations of overall health status and recovery trajectories following emergency laparotomy [19, 39]. While condition specific measures have been found more responsive to changes in health status related to particular condition, generic instruments, such as the EQ-5D capture key aspects of QoL, enable comparisons across different diseases and interventions, and inform cost-effectiveness assessments of interventions to guide their adoption.

Our study has some limitations. First, collecting QoL data following surgery is challenging [39], particularly in distressed patients, and it is, therefore, possible that QoL measurement was more likely to take place in healthier patients [40]. Nevertheless, following missing data imputation using a broad set of patient and surgery characteristics, the QoL estimates we report are likely to provide useful information for the QoL trajectory of patients with similar pre- and post-surgery characteristics. Second, the study did not collect information for further patient characteristics and surgical outcomes impacting patients’ QoL post-emergency abdominal surgery. For example, we could not investigate factors such as nutrition, stoma, delirium or physical and social functioning which are important patient considerations [10, 19]. Third, the post-surgery QoL data and modelling are conditional on patients’ surviving to provide QoL data and, therefore, relevant to survivors at particular time points. The exclusion of the 25 patients with QoL measure shortly after surgery who died during follow up had not material effect on the QoL trajectories and associations reported here. Fourth, while the predictions from the QoL models corresponded well to reported QoL in categories of patients in the study, external data is required to assess the models’ performance further. While this study offers a starting point in characterising QoL after emergency abdominal surgery, further larger studies with even better-characterised patient population and longer duration of follow-up are needed to address some of these limitations. Finally, while preoperative QoL can be an important factor, we did not attempt to quantify preoperative QoL due to data unavailability.

## Conclusions

In summary, we report improving QoL in surviving patient following emergency abdominal surgery with patient and surgery characteristics, hospital care post-surgery and duration since surgery the key determinants of QoL after surgery. The study reports trajectories of QoL and QoL prediction models that can aid discussions between clinicians and patients and guide expectations for improvements in QoL following surgery. They can also aid assessments of interventions to improve emergency abdominal surgery outcomes.

## Supplementary Information

Below is the link to the electronic supplementary material.

Supplementary Material 1

Supplementary Material 2

## Data Availability

Due to information governance restrictions imposed by organisations governing NHS data access, we are unable to share the trial data unless applicants secure the relevant permissions.
