# Supplementary material for: Health-related quality of life after emergency abdominal surgery
Source: World J Emerg Surg. 2025 Sep 2;20:73. doi: 10.1186/s13017-025-00643-1 (PMC12403461; doi:10.1186/s13017-025-00643-1)
Supplement: Supplementary file 1 — Supplementary Material 1 [file 13017_2025_643_MOESM1_ESM.docx]

**Health-related quality of life after emergency abdominal surgery**

Gordeev VS, Assefa E, Pearse R, Edwards M, Mihaylova B.

#### **Supplementary Material**

**Table of contents**

[Table S1. Comorbidities and their ICD-10 codes, as defined by the Charlson comorbidity index 2](#_Toc200920849)

[Table S2. Baseline characteristics of participants with and without QoL measures 3](#_Toc200920850)

[Table S3. Health-related quality of life post-emergency abdominal surgery: multivariable linear regression models (complete case analysis) 4](#_Toc200920851)

[Table S4. Observed and predicted QoL after emergency abdominal surgery 7](#_Toc200920852)

[Figure S1. Schematic of hospital admissions and complications following emergency abdominal surgery 8](#_Toc200920853)

[Figure S2. Participants with EQ-5D data (number of EQ-5D measurements) 9](#_Toc200920854)

[Figure S3. Quality of life after emergency abdominal surgery (A) and after elective abdominal surgery (B), by time since surgery 10](#_Toc200920855)

## Table S1. Comorbidities and their ICD-10 codes, as defined by the Charlson comorbidity index

| Comorbidity |  | ICD-10 |  | Charlson comorbidity  index coefficient |
| --- | --- | --- | --- | --- |
| Myocardial infarction |  | I21.x, I22.x, I25.2 |  | 1 |
| Congestive heart failure |  | I11.0, I13.0, I13.2, I25.5, I42.0, I42.5–I42.9, I43.x, I50.x, P29.0 |  | 1 |
| Peripheral vascular disease |  | I70.x, I71.x, I73.1, I73.8, I73.9, I77.1, I79.0, I79.2, K55.1, K55.8, K55.9, Z95.8, Z95.9 |  | 1 |
| Cerebrovascular disease |  | G45.x, G46.x, H34.0, I60.x–I69.x |  | 1 |
| Dementia |  | F00.x–F03.x, F05.1, G30.x, G31.1 |  | 1 |
| Chronic pulmonary Disease |  | I27.9, J40.x–J47.x, J60.x–J67.x, J68.4, J70.1, J70.3 |  | 1 |
| Rheumatic disease |  | M05.x, M06.x, M31.5, M32.x–M34.x, M35.1, M35.3, M36.0 |  | 1 |
| Peptic ulcer disease |  | K25.x–K28.x |  | 1 |
| Mild liver disease |  | B18.x, K70.0–K70.3, K70.9, K71.3–K71.5, K71.7, K73.x, K74.x, K76.0, K76.2–K76.4, K76.8, K76.9, Z94.4 |  | 1 |
| Diabetes without chronic complication |  | E10.0, E10.1, E10.6, E10.8, E10.9, E11.0, E11.1, E11.6, E11.8, E11.9, E12.0, E12.1, E12.6, E12.8, E12.9, E13.0, E13.1, E13.6, E13.8, E13.9, E14.0, E14.1, E14.6, E14.8, E14.9 |  | 2 |
| Diabetes with chronic complication |  | E10.7, E11.2–E11.5, E11.7, E12.2–E12.5, E12.7, E13.2–E13.5, E13.7, E14.2–E14.5, E14.7 |  | 2 |
| Hemiplegia or paraplegia |  | G04.1, G11.4, G80.1, G80.2, G81.x, G82.x, G83.0–G83.4, G83.9 |  | 2 |
| Renal disease |  | I12.0, I13.1, N03.2–N03.7, N05.2–N05.7, N18.x, N19.x, N25.0, Z49.0–Z49.2, Z94.0, Z99.2 |  | 2 |
| Any malignancy, including lymphoma and leukaemia, except malignant neoplasm of skin |  | C00.x–C26.x, C30.x–C34.x, C37.x–C41.x, C43.x, C45.x–C58.x, C60.x–C76.x, C81.x–C85.x, C88.x,C90.x–C97.x |  | 2 |
| Moderate or severe liver disease |  | I85.0, I85.9, I86.4, I98.2, K70.4, K71.1, K72.1, K72.9, K76.5, K76.6, K76.7 |  | 3 |
| Metastatic solid tumour |  | C77.x–C80.x |  | 6 |
| AIDS/HIV |  | B20.x–B22.x, B24.x |  | 6 |

ICD – international classification of diseases; AIDS – acquired immunodeficiency syndrome; HIV - human immunodeficiency virus. Charlson comorbidity index classification: no comorbidity, mild (1-2 score), moderate (3-4 score), severe (≥5 score).

## Table S2. Baseline characteristics of participants with and without QoL measures

|  | **Short-term (Up to 2 months post-surgery)**  **(n=686)** | | | | |  | **Medium- to long-term (3 to 8 months post-surgery)**  **(n=648)** | | | |
| --- | --- | --- | --- | --- | --- | --- | --- | --- | --- | --- |
|  | **Participants with QoL measure**  **(n=664)** | | **Participants missing QoL measure**  **(n=22)** | |  | **Participants with QoL measure**  **(n=506)** | | **Participants missing QoL measure**  **(n=142 ^b^ )** | |  |
|  | **N** | **(%)** | **N** | **(%)** | ***p*-value** | **N** | **(%)** | **N** | **(%)** | ***p*-value** |
| **Sex** |  |  |  |  | 0.364 |  |  |  |  | 0.934 |
| Male | 327 | (49.2) | 13 | (59.1) |  | 251 | (49.6) | 71 | (50) |  |
| Female | 337 | (50.8) | 9 | (40.9) |  | 255 | (50.4) | 71 | (50) |  |
| **Age, Mean (SD)** |  |  |  |  | 0.770 |  |  |  |  | 0.070 |
| <60 years | 202 | (30.4) | 7 | (31.8) |  | 152 | (30.0) | 56 | (39.4) |  |
| 60-69 years | 159 | (23.9) | 7 | (31.8) |  | 129 | (25.5) | 28 | (19.7) |  |
| 70-79 years | 176 | (26.5) | 4 | (18.2) |  | 137 | (27.1) | 29 | (20.4) |  |
| 80+ years | 127 | (19.1) | 4 | (18.2) |  | 88 | (17.4) | 29 | (20.4) |  |
| **Comorbidity, Charlson Comorbidity Index** |  |  |  |  | 0.286 |  |  |  |  | 0.103 |
| No comorbidity | 292 | (46.5) | 8 | (44.4) |  | 232 | (49.2) | 52 | (38.2) |  |
| Mild | 246 | (39.2) | 10 | (55.6) |  | 179 | (37.9) | 65 | (47.8) |  |
| Moderate | 66 | (10.5) | 0 | (0.0) |  | 45 | (9.5) | 12 | (8.8) |  |
| Severe | 24 | (3.8) | 0 | (0.0) |  | 16 | (3.4) | 7 | (5.1) |  |
| **ASA physical status** |  |  |  |  | 0.378 |  |  |  |  | 0.175 |
| Normal healthy patient/ Mild systemic disease | 275 | (41.4) | 13 | (59.1) |  | 224 | (44.3) | 59 | (41.5) |  |
| Severe systemic disease | 265 | (39.9) | 7 | (31.8) |  | 196 | (38.7) | 57 | (40.1) |  |
| Severe systemic disease, life threatening | 114 | (17.2) | 2 | (9.1) |  | 81 | (16.0) | 21 | (14.8) |  |
| Moribund | 10 | (1.5) | 0 | (0.0) |  | 5 | (1.0) | 5 | (3.5) |  |
| **Preoperative risk of mortality** |  |  |  |  | 0.990 |  |  |  |  | 0.550 |
| Low (<5%) | 152 | (22.9) | 5 | (22.7) |  | 114 | (22.5) | 38 | (26.8) |  |
| High (>=5%) | 262 | (39.5) | 9 | (40.9) |  | 197 | (38.9) | 54 | (38.0) |  |
| Not documented | 250 | (37.7) | 8 | (36.4) |  | 195 | (38.5) | 50 | (35.2) |  |

^a^ p-value for Pearson’s chi-squared test; ^b^ excluding participants who died by day 120

## Table S3. Health-related quality of life post-emergency abdominal surgery: multivariable linear regression models (complete case analysis)

|  | **Model 1:**  **Short-term QoL (up to 2 months post-surgery)** | | | **Model 2a:**  **Medium- to long-term QoL (3 to 8 months post-surgery) with patient and clinical pre-op characteristics** | | | **Model 2b:**  **Medium to long-term QoL (3 to 8 months post-surgery) including post-op characteristics** | | |
| --- | --- | --- | --- | --- | --- | --- | --- | --- | --- |
|  | **632 observations from 627 participants** | | | **1008 observations from 553 participants** | | | **944 observations from 519 participants** | | |
|  | **Coef.** | **(SE)** | ***p*-value** | **Coef.** | **(SE)** | ***p*-value** | **Coef.** | **(SE)** | ***p*-value** |
| **Constant** | 0.396 | (0.051)^a^ | <0.001 | 0.786 | (0.035)^b^ | <0.001 | 0.831 | (0.042)^c^ | <0.001 |
| **Sex (Reference: male)** |  |  |  |  |  |  |  |  |  |
| Female | -0.127 | (0.037) | 0.001 | -0.062 | (0.024) | 0.009 | -0.047 | (0.025) | 0.06 |
| **Age (Reference: <60 years)** |  |  | 0.09 ^d^ |  |  | 0.17 ^d^ |  |  | 0.28 ^d^ |
| 60-69 years | 0.029 | (0.051) | 0.58 | 0.034 | (0.031) | 0.27 | 0.044 | (0.031) | 0.16 |
| 70-79 years | 0.067 | (0.052) | 0.20 | 0.009 | (0.033) | 0.79 | 0.019 | (0.034) | 0.56 |
| 80+ years | 0.093 | (0.059) | 0.11 | 0.068 | (0.038) | 0.07 | 0.054 | (0.039) | 0.16 |
| **Comorbidity, Charlson Comorbidity Index (Reference: No comorbidity)** |  |  | 0.04 ^d^ |  |  | 0.04 ^d^ |  |  | 0.03 ^d^ |
| Mild | -0.067 | (0.042) | 0.11 | -0.036 | (0.026) | 0.16 | -0.042 | (0.027) | 0.12 |
| Moderate | -0.102 | (0.065) | 0.12 | -0.103 | (0.053) | 0.05 | -0.129 | (0.054) | 0.02 |
| Severe | -0.134 | (0.100) | 0.18 | -0.084 | (0.088) | 0.34 | -0.062 | (0.088) | 0.48 |
| **ASA physical status (Reference: Normal healthy patient/ Mild systemic disease)** |  |  | 0.87 ^d^ |  |  | 0.007 ^d^ |  |  | 0.07 ^d^ |
| Severe systemic disease | 0.023 | (0.047) | 0.62 | -0.071 | (0.028) | 0.01 | -0.053 | (0.029) | 0.07 |
| Severe systemic disease, life threatening | -0.007 | (0.063) | 0.91 | -0.122 | (0.043) | 0.004 | -0.086 | (0.044) | 0.05 |
| Moribund | 0.084 | (0.144) | 0.56 | -0.063 | (0.121) | 0.60 | -0.036 | (0.113) | 0.75 |
| **Preoperative risk of mortality (Reference: Low (<5%))** |  |  | 0.01 ^d^ |  |  | 0.12 ^d^ |  |  | 0.22 ^d^ |
| High (>=5%) | -0.136 | (0.053) | 0.01 | -0.042 | (0.032) | 0.20 | -0.018 | (0.033) | 0.59 |
| Not documented | -0.130 | (0.048) | 0.007 | 0.022 | (0.028) | 0.44 | 0.034 | (0.029) | 0.24 |
| **Indication for surgery (Reference: Obstruction)** |  |  | 0.59 ^d^ |  |  | 0.05 ^d^ |  |  | 0.41 ^d^ |
| Haemorrhage | 0.030 | (0.118) | 0.80 | -0.008 | (0.062) | 0.89 | -0.048 | (0.063) | 0.45 |
| Sepsis | -0.063 | (0.043) | 0.14 | 0.060 | (0.024) | 0.01 | 0.038 | (0.028) | 0.18 |
| Ischaemia | -0.063 | (0.068) | 0.35 | -0.016 | (0.052) | 0.76 | 0.010 | (0.052) | 0.85 |
| Other | -0.033 | (0.078) | 0.68 | -0.054 | (0.057) | 0.35 | -0.030 | (0.06) | 0.61 |
| **Duration between index surgery and QoL measurement (Reference: 3-4 months)** |  |  |  |  |  | <0.001 ^d^ |  |  | 0.02 ^d^ |
| 5-6 months |  |  |  | -0.0001 | (0.035) | 0.99 | 0.019 | (0.033) | 0.56 |
| 7-8 months |  |  |  | 0.057 | (0.015) | <0.001 | 0.038 | (0.015) | 0.02 |
| **Index surgery location (Reference: Large intestine)** |  |  |  |  |  |  |  |  | 0.03 ^d^ |
| Small intestine |  |  |  |  |  |  | 0.010 | (0.036) | 0.78 |
| Small or large intestine |  |  |  |  |  |  | -0.082 | (0.063) | 0.19 |
| Stomach |  |  |  |  |  |  | 0.095 | (0.040) | 0.02 |
| Other (abdominal cavity/wall) |  |  |  |  |  |  | -0.030 | (0.030) | 0.32 |
| **Discharge destination after index surgery (Reference: Ward)** |  |  |  |  |  |  |  |  |  |
| ICU/HDU |  |  |  |  |  |  | -0.010 | (0.028) | 0.72 |
| **Length of hospital stay after surgery (Reference: <=10 days)** |  |  |  |  |  |  |  |  | 0.002 ^d^ |
| 11-14 days |  |  |  |  |  |  | -0.007 | (0.033) | 0.84 |
| 15-21 days |  |  |  |  |  |  | -0.038 | (0.031) | 0.21 |
| 21+ days |  |  |  |  |  |  | -0.104 | (0.035) | 0.003 |
| **Length of hospital stay within 30 days before QoL measurement** |  |  |  |  |  |  | -0.026 | (0.006) | <0.001 |
| R-squared | 0.049 |  |  | 0.077 |  |  | 0.144 |  |  |
| Prob > F | 0.01 |  |  | <0.001 |  |  | <0.001 |  |  |

^a^ Short-term QoL for a reference patient: a male patient of age 40-59 years, assessed to be ASA class I or II (normal healthy or mild systemic disease) with low (<5%) preoperative risk of mortality and obstruction as indication for surgery

^b^ Medium to long-term QoL for a reference patient: a male patient of age 40-59 years, assessed to be ASA class I or II (normal healthy or mild systemic disease) with low (<5%) preoperative risk of mortality, obstruction as indication for surgery and 3-4 months post-surgery

^c^ Medium to long-term QoL for a reference patient: a male patient of age 40-59 years, assessed to be of ASA class I or II (normal healthy or mild systemic disease), with low (<5%) preoperative risk of mortality, obstruction as indication for surgery, main procedure performed on large intestine, discharged to ward post-surgery, stayed 10 days or less in hospital during index surgery, no hospitalisation within 30 days before QoL measurement and 3-4 months post-surgery

^d^ p-value for linear trend test if ordinal or heterogeneity test if nominal variables

R-squared based on Fisher’s z transformation (using ‘mibeta’ Stata command).

QoL, quality of life; Prob, probability; F, F-value

## Table S4. Observed and predicted QoL after emergency abdominal surgery

|  | Short-term QoL (1-2 months post-surgery) | | | | | | | Medium- to long-term QoL (3 to 8 months post-surgery) | | | | | | | | | |
| --- | --- | --- | --- | --- | --- | --- | --- | --- | --- | --- | --- | --- | --- | --- | --- | --- | --- |
|  | Reported | | | Predicted (model 1) | | | Reported | | | | Predicted (model 2a) | | | Predicted (model 2b) | | |  |
|  | N | Mean | (SE) | N | Mean | (SE) | N | | Mean | (SE) | N | Mean | (SE) | N | Mean | (SE) |  |
| **Overall** | 669 | 0.21 | (0.018) | 632 | 0.22 | (0.005) | 1,079 | | 0.74 | (0.009) | 1,008 | 0.73 | (0.003) | 944 | 0.73 | (0.003) |  |
|  |  |  |  |  |  |  |  | |  |  |  |  |  |  |  |  |  |
| **Sex** |  |  |  |  |  |  |  | |  |  |  |  |  |  |  |  |  |
| Male | 329 | 0.27 | (0.025) | 313 | 0.27 | (0.007) | 533 | | 0.76 | (0.013) | 499 | 0.76 | (0.004) | 467 | 0.76 | (0.004) |  |
| Female | 340 | 0.16 | (0.025) | 319 | 0.16 | (0.007) | 546 | | 0.72 | (0.014) | 509 | 0.71 | (0.004) | 477 | 0.71 | (0.004) |  |
|  |  |  |  |  |  |  |  | |  |  |  |  |  |  |  |  |  |
| **Age** |  |  |  |  |  |  |  | |  |  |  |  |  |  |  |  |  |
| <60 years | 205 | 0.19 | (0.032) | 197 | 0.20 | (0.009) | 331 | | 0.75 | (0.016) | 315 | 0.75 | (0.005) | 290 | 0.75 | (0.005) |  |
| 60-69 years | 160 | 0.20 | (0.036) | 151 | 0.20 | (0.010) | 283 | | 0.75 | (0.018) | 260 | 0.74 | (0.006) | 244 | 0.74 | (0.006) |  |
| 70-79 years | 176 | 0.24 | (0.035) | 162 | 0.24 | (0.010) | 277 | | 0.72 | (0.020) | 254 | 0.72 | (0.007) | 240 | 0.72 | (0.007) |  |
| 80+ years | 128 | 0.24 | (0.040) | 122 | 0.24 | (0.011) | 188 | | 0.73 | (0.021) | 179 | 0.71 | (0.008) | 170 | 0.72 | (0.008) |  |
|  |  |  |  |  |  |  |  | |  |  |  |  |  |  |  |  |  |
| **Comorbidity, CCI** |  |  |  |  |  |  |  | |  |  |  |  |  |  |  |  |  |
| No comorbidity | 294 | 0.25 | (0.026) | 293 | 0.26 | (0.007) | 486 | | 0.77 | (0.012) | 484 | 0.77 | (0.004) | 446 | 0.77 | (0.004) |  |
| Mild | 249 | 0.18 | (0.030) | 249 | 0.19 | (0.009) | 394 | | 0.72 | (0.016) | 394 | 0.72 | (0.005) | 370 | 0.72 | (0.005) |  |
| Moderate | 66 | 0.17 | (0.057) | 66 | 0.17 | (0.016) | 94 | | 0.63 | (0.041) | 94 | 0.63 | (0.013) | 92 | 0.63 | (0.013) |  |
| Severe | 24 | 0.15 | (0.094) | 24 | 0.16 | (0.027) | 36 | | 0.66 | (0.065) | 36 | 0.66 | (0.021) | 36 | 0.67 | (0.021) |  |
|  |  |  |  |  |  |  |  | |  |  |  |  |  |  |  |  |  |
| **Duration between index surgery and QoL measurement** |  |  |  |  |  |  |  | |  |  |  |  |  |  |  |  |  |
| 3-4 months |  |  |  |  |  |  | 507 | | 0.71 | (0.013) | 473 | 0.71 | (0.004) | 445 | 0.71 | (0.004) |  |
| 5-6 months |  |  |  |  |  |  | 120 | | 0.71 | (0.032) | 114 | 0.71 | (0.009) | 103 | 0.70 | (0.008) |  |
| 7-8 months |  |  |  |  |  |  | 452 | | 0.77 | (0.014) | 421 | 0.77 | (0.004) | 396 | 0.77 | (0.004) |  |

CCI, Charlson Comorbidity Index; QoL, quality of life; SE, standard error

## Figure S1. Schematic of hospital admissions and complications following emergency abdominal surgery


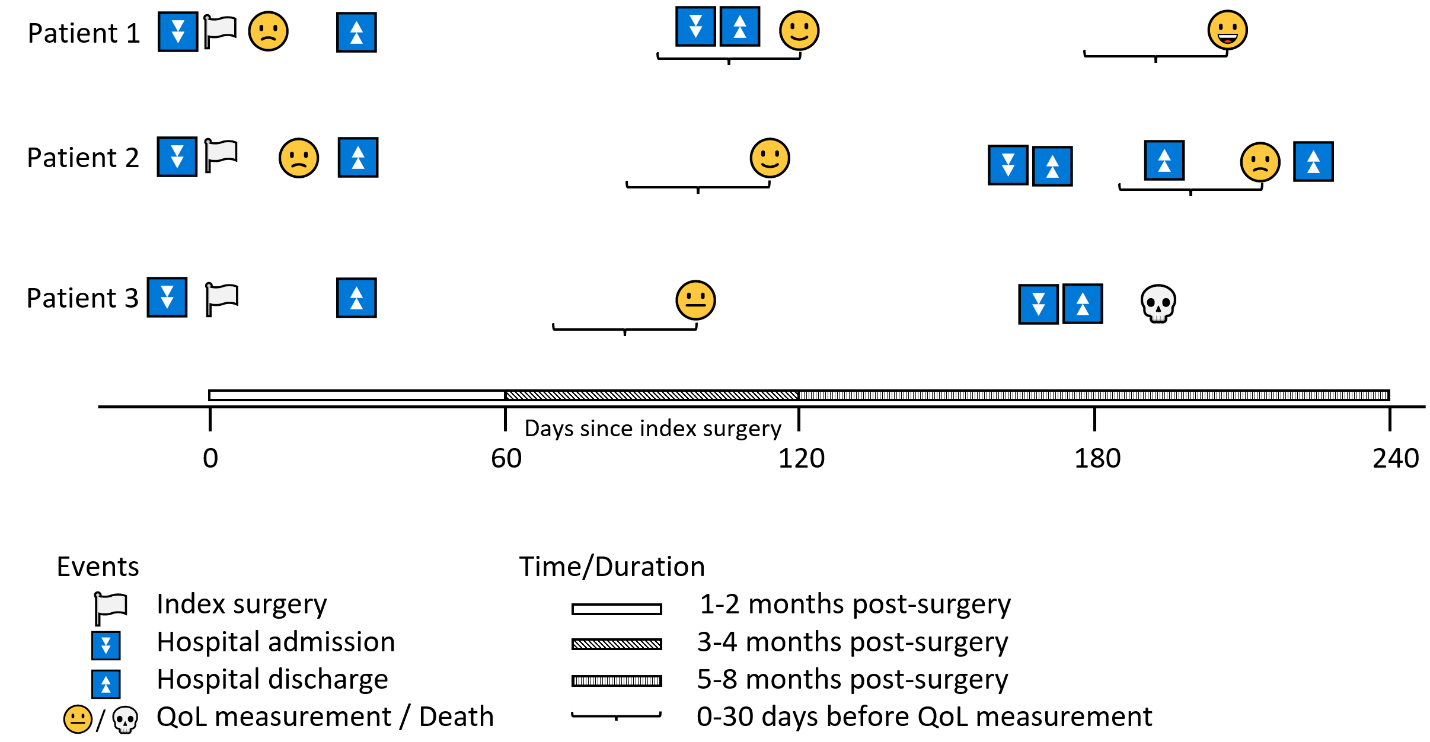


QoL – the quality of life. Emoji expressions are used for illustrative purposes only to represent hypothetical patient cases, where some patients experience no problems and report good QoL, and some report poorer QoL due to various reasons (i.e., complications, multimorbidity).

## Figure S2. Participants with EQ-5D data (number of EQ-5D measurements)


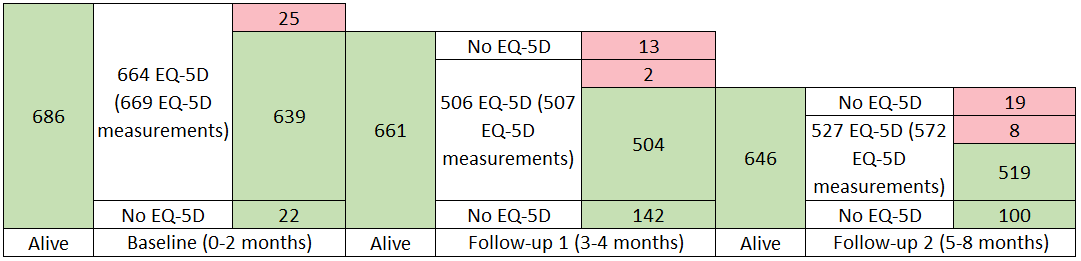


Notes: green – alive, red – died; EQ5D – the quality of life measurement present. Baseline – within 2 months post-surgery; follow-up 1 - 3-4 months post-surgery); follow-up 2 - 5-8 months post-surgery

## Figure S3. Quality of life after emergency abdominal surgery (A) and after elective abdominal surgery (B), by time since surgery


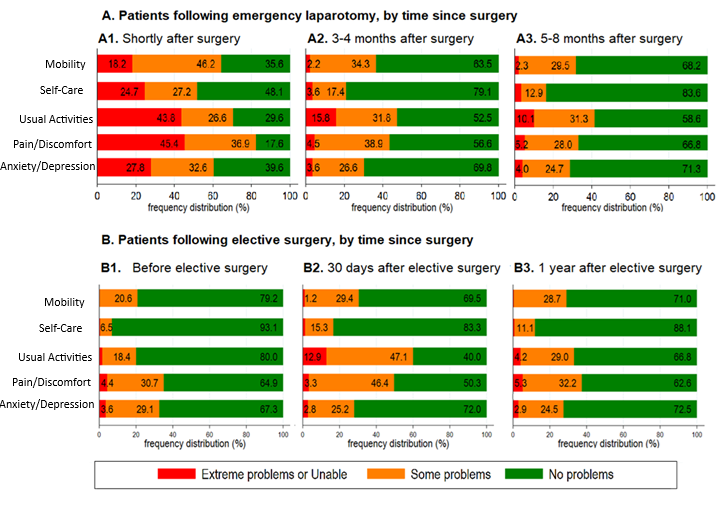


EQ-5D-3L descriptive system comprises five domains: mobility, self-care, usual activities, pain/discomfort, and anxiety/depression. Each domain has three levels: no problems, some problems, and extreme problems.  Panel A: EPOCH trial data. Panel B: PRISM - Prevention of Respiratory Insufficiency after Surgical Management (PRISM) trial data. For PRISM (Panel B) chart: before surgery (n=2811), 30 days after elective surgery (n=2653), and one year after elective surgery (n=2262)
